# EMG, Rate of Perceived Exertion, Pain, Tolerability and Possible Adverse Effects of a Knee Extensor Exercise with Progressive Elastic Resistance in Patients with Severe Haemophilia

**DOI:** 10.3390/jcm9092801

**Published:** 2020-08-30

**Authors:** Joaquín Calatayud, Jonás Navarro-Navarro, Juan J. Carrasco, Sofía Pérez-Alenda, Carlos Cruz-Montecinos, Lars L. Andersen, Felipe Querol-Giner, José Casaña

**Affiliations:** 1 Exercise Intervention for Health Research Group (EXINH-RG), Department of Physiotherapy, University of Valencia, 46010 Valencia, Spain; joaquin.calatayud@uv.es (J.C.); jonasnavarro7@hotmail.com (J.N.-N.); jose.casana@uv.es (J.C.); 2 National Research Centre for the Working Environment, 2100 Copenhagen, Denmark; lla@nfa.dk; 3 Physiotherapy in Motion Multispeciality Research Group (PTinMOTION), Department of Physiotherapy, University of Valencia, 46010 Valencia, Spain; sofia.perez-alenda@uv.es (S.P.-A.); ccmkine@gmail.com (C.C.-M.); felipe.querol-giner@uv.es (F.Q.-G.); 4 Intelligent Data Analysis Laboratory, University of Valencia, 46100 Valencia, Spain; 5 Laboratory of Clinical Biomechanics, Department of Physical Therapy, Faculty of Medicine, University of Chile, 8380453 Santiago, Chile; 6 Sport Sciences, Department of Health Science and Technology, Aalborg University, 9220 Aalborg East, Denmark

**Keywords:** haemophilic arthropathy, strength, exercise therapy, quadriceps, muscle activity

## Abstract

In people with haemophilia (PWH), elastic band training is considered an optimal option, even though the literature is scarce. The aim was to evaluate normalized electromyographic amplitude (nEMG), rate of perceived exertion (RPE), pain, tolerability, and possible adverse effects during the knee extension exercise using multiple elastic resistance intensities in PWH. During a single session, 14 severe PWH undergoing prophylactic treatment performed knee extensions without resistance and with different intensity levels of elastic resistance. nEMG was measured for the knee extensors and participants rated their RPE, tolerability and pain intensity after each condition. Patients had to report the possible adverse effects after the session. In most of the cases, an nEMG increase is only evidenced after increasing the resistance by two to three levels. Significant associations were found between RPE and the nEMG (*ρ* = 0.61), as well as between the elastic resistance level and nEMG (*ρ* = 0.69) and RPE (*ρ* = 0.71). All conditions were generally tolerated, without increased pain, and no adverse effects were reported. A wide variety of elastic resistance intensities during the knee extension are safe, tolerated, and do not increase knee pain in the majority of severe PWH undergoing prophylactic treatment.

## 1. Introduction

Haemophilia is an inherited recessive pathology of the X chromosome that affects blood coagulation. Therefore, women are carriers and men are the most affected gender, although women can also develop other types of coagulopathies [1]. Haemophilia is caused by a factor VIII (type A) or IX (type B) coagulation deficiency, which have incidences of 1/5000 and 1/25,000 in men, respectively [2]. 

This bleeding disorder causes delayed coagulation after injury, with spontaneous joint (70–80% of cases) and muscle (10–20% of cases) bleedings even during low-impact activities [3]. Bleedings have very serious consequences on the musculoskeletal system, impairing functionality and quality of life in this population, with the knee as the most commonly affected joint [4]. This is especially relevant considering that around 90% of severe people with hemophilia (PWH) will develop arthropathy in the target joints during their second or third decade of life [5].

Strength training is recommended in PWH to optimize function, and decrease pain and the frequency and severity of bleeding episodes in spite of the literature’s scarcity [6,7,8]. Different types of resistance can be used to perform strength training, which can induce specific physiological responses [9]. Among all types of resistance, recent studies point out that training with elastic bands is an optimal option due to its economic accessibility, portability, safety and effectiveness [10,11,12]. Specifically, two studies conducted in PWH have shown that, in general, elastic bands provide similar muscle activation to other traditional methods, such as machines [10] or free weights [11], in lower and upper limbs, respectively. However, there are no other studies using elastic resistance in PWH, while their use in other chronic pathologies is more common [13]. For example, how muscle activation levels vary by increasing elastic resistance has not been analyzed. Although the progression of intensity with elastic bands has traditionally been determined by colors and levels established by each brand, this might not be equivalent to a real increase in muscle activation, which is an indicator of the muscular excitement [14]. This might be relevant to prescribing a safe and effective intensity progression. Furthermore, it is unknown how multiple increments of elastic resistance affect pain and patient tolerance, which should be considered when prescribing therapeutic exercise in PWH due to their bleeding risk.

In order to assess and control exercise intensity with the elastic bands, which is crucial to obtaining the desired adaptations and reducing injury risk [15], different approaches can be used. In this sense, the rate of perceived exertion (RPE) through the Borg CR10 scale is one of the most widely used, accessible and accurate markers for quantifying perceived intensity [16]. For instance, a previous study concluded that the RPE during the first three repetitions of a free-weight exercise was a good indicator to differentiate between light–moderate loads (<70% of the one repetition maximum (1 RM)) and high loads (>70% 1 RM), 6 out of 10 being the reference value to enter into the category of high loads [17]. However, no study has evaluated the relationship between the RPE, the use of multiple elastic resistances and the muscle activation. This could be very useful in rapidly assessing and prescribing intensity in PWH without reaching a maximum effort, with the corresponding increased risk that this entails, and without generating additional fatigue. In addition, it would allow for estimating the activation value generated with each RPE increase, helping to establish adequate progressions during exercise.

The main objective of the following study was to evaluate muscle activation, RPE, pain intensity, tolerability and possible adverse effects during knee extension exercise using multiple intensities of elastic resistance in PWH. As a secondary objective, we wanted to evaluate the relationship between the RPE, muscle activity and the elastic resistance level.

## 2. Experimental Section

### 2.1. Participants

Patients over 18 years of age, diagnosed with severe haemophilia and undergoing prophylactic treatment were candidates for the present study.

The exclusion criteria were as follows: patients who received prosthetic surgery in the previous year, patients who had muscle or joint bleeding in the last 3 months, and patients with any other medical condition in which exercise could be contraindicated.

The study took place at the University of Valencia (Valencia, Spain) during March 2020. All the participants were informed about the objective of the study and, in this way, we obtained their respective informed consents. 

The study conformed to the Declaration of Helsinki and was approved by the local ethics committee (reference number: H1461147538087). This article adheres to the STROBE guidelines.

### 2.2. Procedure

The following clinical variables were collected from patients’ medical histories: type of haemophilia and severity, and prophylactic treatment. 

Each participant carried out one experimental session. The participants were not allowed to eat, drink or take stimulants (such as caffeine) 2 h before the session and were not allowed to perform more intense physical activity than daily life activities 24 h prior to the measurement. They were also recommended to sleep a minimum of 7–8 h the night before the assessments. All measurements were made by the same two researchers and were conducted in the same facility.

The participants attended the experimental session 2 to 4 h after receiving their routine coagulation factor prophylaxis treatment. In the experimental session, height (determined with a stadiometer; model IP0955 (Invicta Plastics Ltd., Leicester, UK)) and body mass (determined with a body composition analyzer; model BF-350 (Tanita, Tokyo, Japan)) were obtained. The degree of hemophilic arthropathy was clinically evaluated using the Hemophilia Joint Health Score 2.1. This instrument scores each joint (both knees, ankles and elbows) from 0 to 20 points, with higher scores reflecting worse conditions (having a maximum score of 120 points) [18].

Subsequently, participants answered a short questionnaire about leisure-time physical activity [19] and their resistance training experience. Afterwards, the electromyography (EMG) protocol started with the preparation of participants’ skin, followed by electrode placement, maximum voluntary isometric contractions (MVIC) and the performance of the different exercise conditions.

Hair was removed from the skin overlying the muscles of interest, and the skin was then cleaned by rubbing with cotton wool dipped in alcohol for the subsequent electrode placement. Electrodes were placed on the rectus femoris (RF), vastus lateralis (VL) and vastus medialis (VM) muscles on the dominant leg following the SENIAM (Surface EMG for Non-Invasive Assessment of Muscles) recommendations [20]. The dominant side was determined by the question: “Which leg do you prefer to hit a ball with?” Specifically, electrodes for the RF were fixed at 50% of the distance between the anterior superior iliac spine and the superior part of the patella in the same direction of that line. For the VM, the electrodes were placed at 80% of the line between the anterior superior iliac spine and the joint space in front of the anterior border of the medial ligament of the knee, with a perpendicular orientation to that line. For the VL, the electrodes were located at two-thirds of the line that connects the anterior superior iliac spine with the lateral side of the patella following the orientation of the muscle fibers. Pregelled bipolar silver/silver chloride (Ag/AgCl) surface disk-shape electrodes (Blue Sensor N-00-S; Ambu A/S, Ballerup, Denmark) with an electrode size of 44.8 × 22 mm and a measuring area of 95 mm^2^ were placed with an inter-electrode distance of 2 cm. One reference electrode was positioned a finger-length from the other electrodes for the RF muscle and above the patella for the VL and VM muscles, according to manufacturer’s specifications. EMG data was assessed using Shimmer3 sensors (Shimmer Sensing, Dublin, Ireland), with the signal processed through “The mDurance system” software (mDurance solutions, Granada, Spain). All signals were acquired at a sampling frequency of 1024 Hz with a bandwidth of 8 to 500 Hz so as to avoid aliasing. Before starting each exercise condition, we checked the offset values for each channel to ensure that they were within a ±2 μV range of 0 μV. 

At the beginning of the experimental session, the participants performed a submaximal isometric contraction as a practical trial to ensure that they had understood the task. Subsequently, they were asked to complete two MVICs with a 1 min rest between trials. The patients performed a 2 s progressive contraction and then maintained a maximum contraction for the next 3 s. Verbal encouragement was provided to motivate all participants to reach their maximal effort.

The position during the evaluation of the MVICs was based on standardized muscle testing procedures for RF [21] and was performed against a fixed resistance. Specifically, the knee extension was performed with the participant seated with 70° of knee flexion and 110° of hip flexion. In addition, patients maintained a neutral dorsiflexion of the ankle at 90°. 

**Figure 1:**
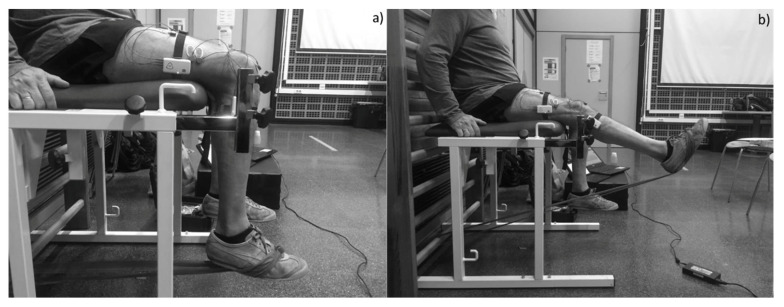
Knee extension exercise with elastic band. (**a**) initial phase of the exercise; (**b**) final phase of the exercise.

After a 2 min break, the knee extension exercise was performed (Figure 1). This was completed in the same place and with the same described position, with 8 different conditions randomly (by using a computer-based “random number generator”) performed and separated by 2 min of rest between them. Seven of these conditions were conducted with different elastic resistances (Thera-Band CLX; The Hygenic Corp, Akron, OH, USA) according to their color (yellow, red, green, blue, black, silver and gold) and a condition was conducted without external resistance (body weight). According to the elastic band’s manufacturer, there is 20% resistance difference between the different bands, except between the two hardest levels (silver and gold), where there is an increase of 30% resistance. Specifically, the different resistances according to the elastic band’s manufacturer at 100% elongation are 1.36 kg, 1.67 kg, 2.09 kg, 2.63 kg, 3.31 kg, 4.63 kg and 6.44 kg for the yellow, red, green, blue, black, silver and gold bands, respectively.

The band length was 1.9 m and they were pre-stretched to 50% of their size before performing the exercise to achieve an appropriate intensity. 

The exercise was performed with the participant’s available range of motion. Participants were asked to move their body and trunk as little as possible and to perform the exercise smoothly without stops or accelerations. For this purpose, a metronome was used with a speed of 1.5 s for concentric contraction and 1.5 s for eccentric contraction, with a beep sound at the change of each phase. If they were not able to perform the exercise with the correct technique, the attempt was canceled and repeated again. If the patient was not able to reach the three repetitions during a certain condition, the experimental session finished. After performing each condition, a researcher showed the Borg CR10 scale to the patients and they had to report their RPE. Then, they were asked about their pain intensity in the knee used to perform the exercise with an 11-point numerical pain scale. This scale showed excellent test–retest reliability, with an Intraclass Correlation Coefficient (ICC) of 0.83 [22]. Later, patients were asked about the degree of perceived tolerability with a five-point scale (i.e., very well tolerated, tolerated, neutral, not well tolerated and not tolerated). Finally, 24–48 h later, the participants were asked about any possible adverse effect (i.e., bleeding or pain). Furthermore, they were required to report any adverse effect they might feel during the week after the session.

### 2.3. EMG Processing

EMG data processing was performed using custom-made algorithms implemented in MATLAB (version R2015a; The MathWorks, Inc., Natick, MA, USA) software. For the analysis, all raw EMG signals obtained during the exercises were digitally filtered with Butterworth fourth-order high-pass filtering at 10 Hz and a moving root-mean-square (RMS). The RMS routine was performed using a smoothing filter/window of 500 milliseconds (250 milliseconds backward and 250 milliseconds forward from each data point) across the entire signal (i.e., across all contractions). In each of the muscles and for each level of exercise intensity, an RMS peak from each of the contractions was obtained (i.e., total of three RMS peaks). These three RMS peaks were averaged and the value obtained was then normalized to the maximum activation value reached at the MVICs.

### 2.4. Statistical Analysis

The analysis was performed with the MATLAB statistical library. The normality of the data was verified with the Shapiro–Wilk test. Descriptive results are shown as the mean (standard deviation) or median (quartile 1–quartile 3). The differences between percentages of RMS-normalized EMG amplitude (nEMG), grouped by the level of exercise intensity, were analyzed using Student’s t tests or Wilcoxon’s tests as a function of normality. Bonferroni correction was applied to avoid Type I error due to multiple comparisons. Since the number of comparisons between the different levels of exercise intensity is 28, the differences with a *p* value lower than 0.0018 (0.05/28) were considered significant. The relationship between the percentages of nEMG (average value of the three muscles) and RPE and the level of intensity was investigated using Spearman’s correlation coefficient (*ρ*). 

## 3. Results

A total of 14 PWH type A undergoing prophylactic treatment voluntarily participated in the study. Demographic and descriptive data for the participants appear in Table 1. 

Most of the participants in this study performed physical activity at least 2 days a week for more than 15 min. The majority of them also performed strength training at least one day a week with moderate intensity. Complete leisure-time physical activity data are shown in Table 2.

**Figure 2:**
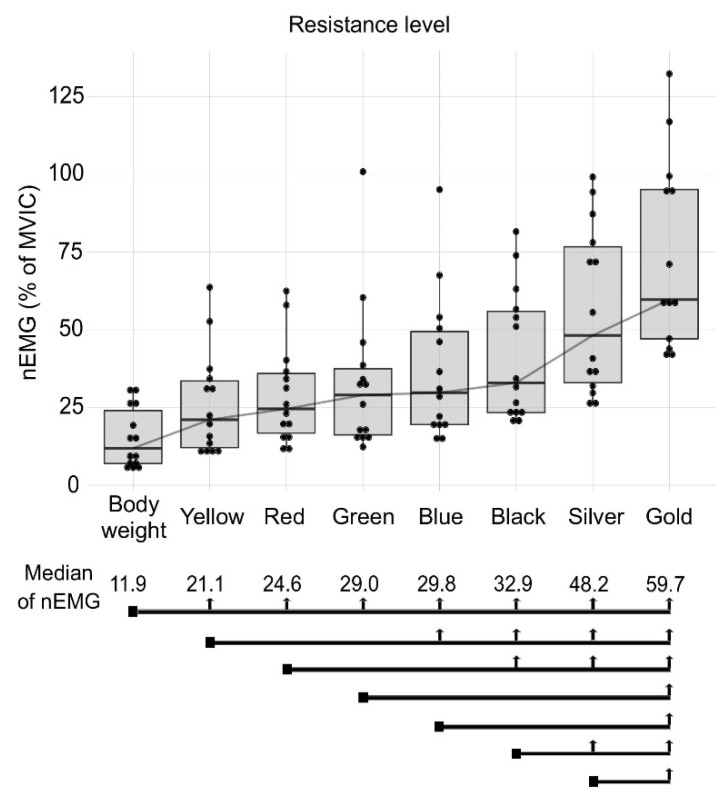
Median muscle activation (Median of nEMG) of RF muscle in the different conditions: body weight, yellow, red, green, blue, black, silver and gold elastic band. The square point at the beginning of the black line marks the compared condition and the vertical arrows show significant differences between conditions.

Figure 2 shows the nEMG of the RF muscle during the performance of the eight conditions. The body weight condition had a median nEMG of 11.9% and reported lower values than all the other elastic band conditions. For the yellow, red, green and blue band conditions, a two-level elastic resistance increase did not represent a real increment in the RF nEMG signal. Finally, the gold band was the condition that provided the highest nEMG in the RF (59.7%), showing significant differences from the other conditions.

**Figure 3:**
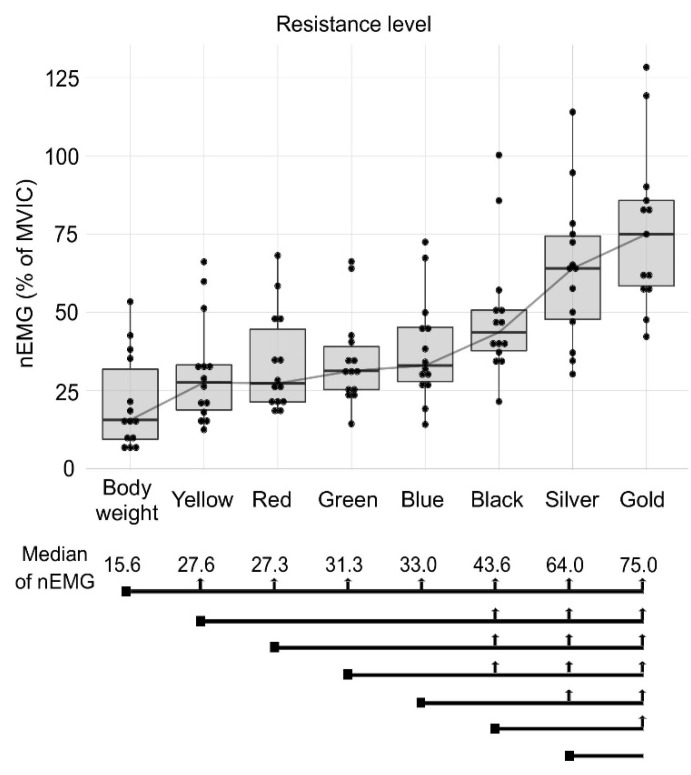
Median muscle activation (Median of nEMG) of the VM muscle for the different conditions: body weight, yellow, red, green, blue, black, silver and gold elastic band. The square point at the beginning of the black line marks the compared condition and the vertical arrows show significant differences between conditions.

Figure 3 shows results of the VM nEMG. The body weight condition had the lowest values of nEMG (15.6%). For the yellow band condition, the three-level increment in elastic resistance did not represent a real increase in the nEMG for the VM muscle. For the red band condition, a two-level increment in elastic resistance did not represent a real nEMG increase for the VM muscle. For all other conditions (green, blue, black and silver bands), a one-level increase in elastic resistance did not mean a real nEMG increase for the VM. The gold band showed greater VM nEMG (75.0%) than the rest of the conditions, except for the silver band. 

**Figure 4:**
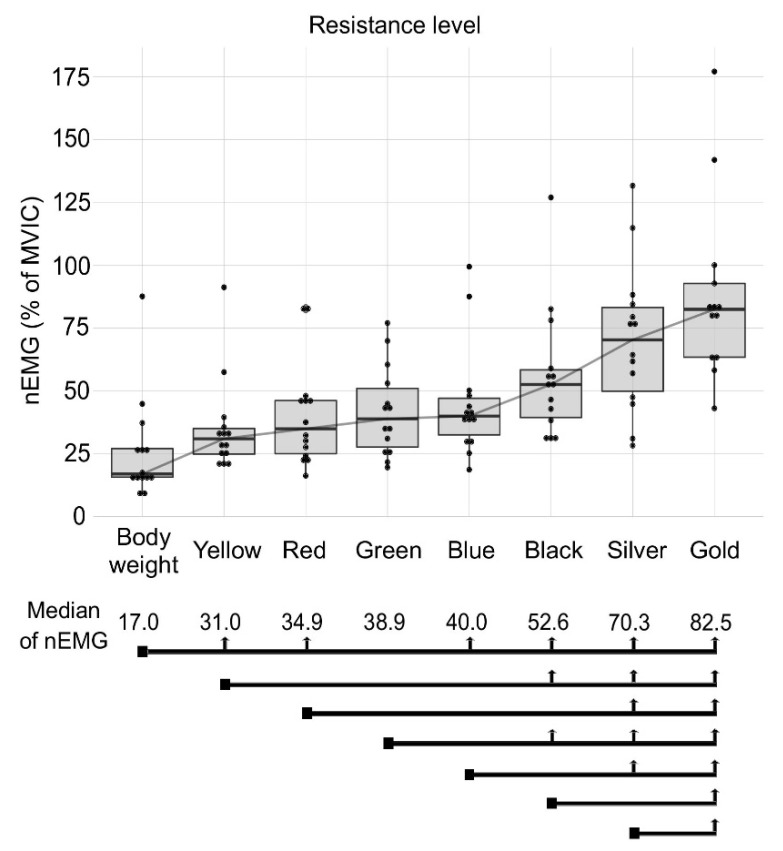
Median muscle activation (Median of nEMG) of the VL muscle for the different conditions: body weight, yellow, red, green, blue, black, silver and gold elastic band. The square point at the beginning of the black line marks the compared condition and the vertical arrows show significant differences between conditions.

Results for VL nEMG are shown in Figure 4. Again, the body weight condition had the lowest values of nEMG (17.0%). For the yellow and red band conditions, a three-level increase in elastic resistance did not increase VL nEMG. For the green, blue and black band conditions, a one-level increment in elastic resistance did not increase VL nEMG. The gold band condition provided a greater VL nEMG (82.5%) than the other conditions.

**Figure 5:**
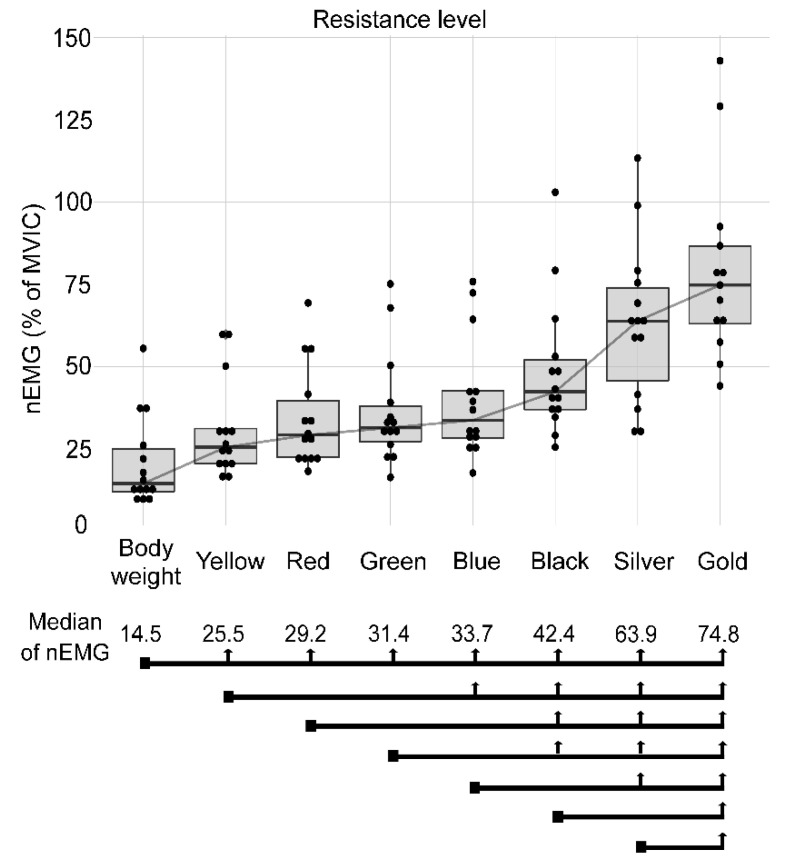
Median muscle activation (median of nEMG) for the different conditions: body weight, yellow, red, green, blue, black, silver and gold elastic band. The square point at the beginning of the black line marks the compared condition and the vertical arrows show significant differences between conditions.

Finally, Figure 5 shows the averaged quadriceps muscle nEMG. The body weight condition had a median nEMG of 14.5%, this being significantly lower than all the other conditions. For the yellow and red band conditions, a two-level increase in elastic resistance did not increase nEMG. For the green, blue and black band conditions, a one-level increment in elastic resistance did not increase nEMG. Finally, the gold band condition provided a greater averaged nEMG (74.8%) than the other conditions.

**Figure 6:**
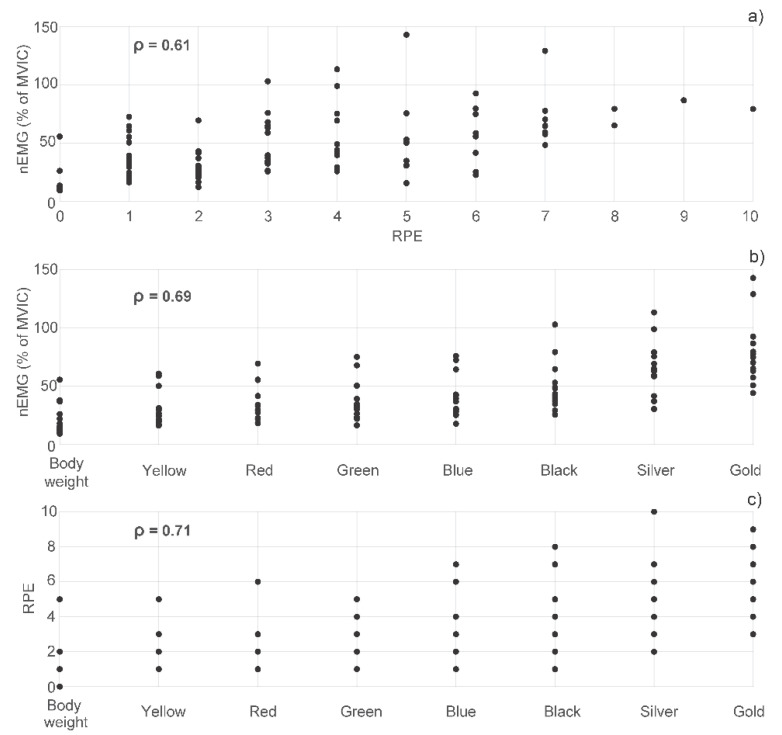
Correlation analysis. The circles represent the nEMG (average value of the three muscles) vs. RPE (**a**), the nEMG vs. the level of intensity (**b**) and the RPE vs. the level of intensity (**c**).

Figure 6 shows the relationship between the averaged nEMG of the knee extensors and RPE (a), as well as between the intensity of the different conditions and the nEMG (b) and RPE (c). Regarding the first, the averaged nEMG presented a medium correlation (*ρ* = 0.61) with the RPE. Regarding the association between intensity and nEMG, the correlation was also medium (*ρ* = 0.69). Finally, the correlation between intensity and RPE was medium–high (*ρ* = 0.71). 

**Figure 7:**
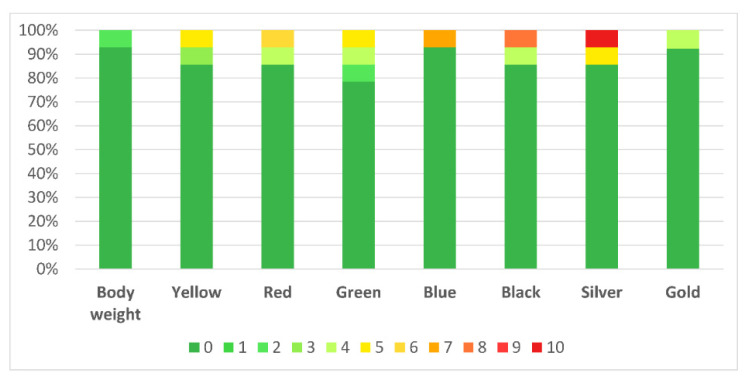
Percentage of intensity-related pain for every condition.

Regarding pain intensity before the session, 10 participants reported 0 pain, 3 participants scored 1–2 and only 1 participant scored 7 in that scale. There were no changes in pain after the session. Regarding the pain caused by each level of elastic resistance, most of the patients reported 0 pain. Figure 7 shows the percentage of intensity-related pain for every condition.

**Figure 8:**
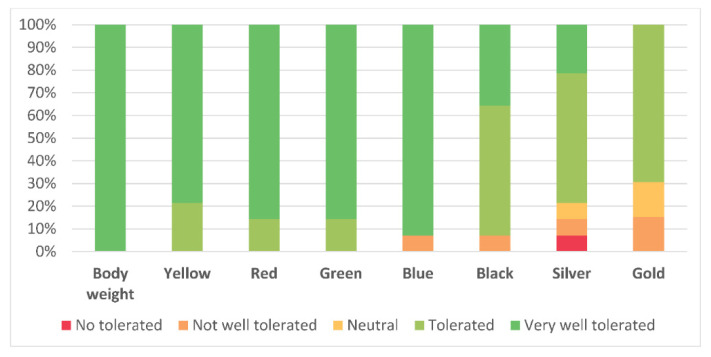
Percentage of intensity-related pain for every condition.

The majority of patients found all the conditions to be “very well tolerated” or “tolerated”. Only one participant did not tolerate the silver band. Figure 8 shows tolerability percentages. 

Finally, no participant reported adverse effects after the experimental session.

## 4. Discussion

The main objective of the following study was to evaluate nEMG, RPE, pain intensity, tolerability and possible adverse effects during knee extension exercise, using different intensities of elastic resistance, in PWH. Evaluating the relationship between the RPE on the aforementioned nEMG and the increase in resistance was the secondary objective. The main findings of the study were as follows: (1) in general, nEMG increased only after increasing 2–3 resistance levels; (2) there were significant relationships between the elastic resistance level and nEMG and RPE, and between the RPE and nEMG; (3) there was general tolerability and an absence of adverse effects. 

Different studies have shown an nEMG increment when a substantial intensity increase occurred in the elastic resistance [23,24]. Skals et al. [23] found nEMG differences between the high (measured as a score of 8 on the Borg CR10 scale) and low (3 on the Borg CR10 scale) intensities for 12 muscle groups during four different exercises in hospitalized patients. Similarly, Brandt et al. [24] found an nEMG increase in 7 of 13 muscles assessed, when using four different intensity levels established by the Borg CR10 scale (low ≤ 2, moderate > 2 < 5, high ≥ 5 < 7 and close to maximum ≥ 7) during hip abduction with an elastic band. Although it seems logical to boost the actual activation by substantially increasing the intensity of the exercise, as occurred in the aforementioned studies, to date, no study has evaluated 8 consecutive resistance levels. Our study shows that there is no consistent nEMG increase at each resistance level. In the same vein, although with a different type of resistance (i.e., free weights), a previous study found a nonlinear muscle activity increase with increasing loads during the squat [25]. Authors reported similar muscle activity in the prime movers for loads between 40% and 60% of 1RM, and between 70% and 90% of 1-RM. Therefore, the increase in elastic resistance corresponding to each color band (according to the manufacturer) does not always correspond to a real nEMG increase in the VL, VM and RF during the knee extension exercise in PWH. In fact, in most of the cases, an nEMG increase is only evidenced after an increase of two to three resistance levels. Consequently, it would be possible to make a more effective exercise progression by using this approach (together with other subjective intensity measures) than by solely using the color as a reference.

In accordance with the above-mentioned results, we found that the RPE increased when elastic resistance increased, although such an increase was not as high and consistent as could be expected. A previous study found a moderate–very strong association between the RPE on the Borg CR10 scale and the nEMG in the main muscles during different shoulder exercises with elastic bands or free weights [26]. Likewise, a strong association was found between the RPE on the OMNI-Resistance Exercise Scale and nEMG during two shoulder exercises with elastic resistance, but only two intensities were used (100% and 150% of elastic band grip width, to achieve 15 repetition maximum (RM)) [27]. Another study [28] recorded the RPE for intensities of 33%, 66% and 100% of the 10 RM during the lunge exercise with elastic resistance, obtaining significant average increases of 1.9 and 1.7 points between them, respectively. However, there are no similar studies associating RPE with nEMG and multiple elastic resistance levels. Such large number of resistance levels could explain the lower association between nEMG and RPE that we found when comparing with the previous studies. Importantly, our results highlight that the RPE could be an imprecise method to distinguish between similar intensities. Nevertheless, the use of the RPE could be useful as a time-efficient first load assessment, avoiding maximal effort and fatigue. However, during a training session, it is likely that a certain number of RM or a submaximal intensity based on the repetitions in reserve are more precise options than the RPE for prescribing intensity, since both are associated with a task failure moment and a feeling of fatigue. Moreover, depending on the RPE of the patient, we could find a balance between the real external load increase and the perceived intensity. This is especially important because it offers the possibility of avoiding intensity-related injuries [29] in a population predisposed to suffering joint bleedings [30,31].

It should be noted that all the elastic resistances were generally “very well tolerated” and that the tolerability slightly decreased as intensity increased, as could be expected. Only one patient was not able to perform the highest available intensity (i.e., gold color), which should be explained by their worsened joint and muscle status. Importantly, no pain increases and no adverse effects were found after the experimental session. This matches with previous studies that have shown a high tolerability of elastic resistance training in patients with arthritis [32] and chronic obstructive pulmonary disease [33], as well as in PWH [10,11]. A high tolerability, together with the possibility of exercising autonomously using the bands, generates greater satisfaction, which is related to higher exercise adherence [34,35]. 

The present study has both strengths and limitations. The fact that the subjects did not report adverse effects after the experimental session does not mean that some minor events (e.g., subclinical haemarthrosis or a painless ROM limitation) did not exist. This could be relevant for long-term training, and future longitudinal studies are needed. However, it seems that a tailored haematological treatment can help avoid major adverse effects after performing moderate intensity exercises in a single session [10,11], or even during a program with two weekly sessions for two months [12]. Despite the fact that it has been used in previous works [10,11], the lack of validation of the tolerability scale is a limitation. Since we did not include a control group, we do not know whether our results in PWH may differ from those achieved in healthy people, and future studies should corroborate this. We did not measure force output, which could provide additional information to corroborate manufacturer data. Moreover, measuring pain intensity and adverse events in a single exercise session is a limitation, and future longer studies should corroborate our results. However, our approach is appropriate as a first step, in order to avoid future complications that could worsen the patient’s condition. Future studies should explore the effects of a longer intervention over time on safety, nEMG, strength, hypertrophy, quality of life, tolerance and pain. 

## 5. Conclusions

A wide variety of elastic resistance levels during the knee extension exercise are safe, well tolerated and do not increase knee pain in PWH undergoing prophylactic treatment. In most of the cases, an nEMG increase is only evidenced after increasing resistance by two to three levels. RPE and nEMG, as well as the elastic resistance level and nEMG and RPE, are significantly associated. The present results may help to prescribe elastic resistance in PWH. However, therapists should be aware of individual tolerability.

## Figures and Tables

**Table 1:** Demographic and descriptive data.

	**Mean**	**SD**
Age (years)	39.0	9.8
Height (cm)	174.6	9.2
Body mass (kg)	81.0	14.9
FVIII dose (IU/Kg)	24.5	10.0
	**Median**	**Q1–Q3**
HJHS ^1^ dominant knee	0.0	0.0–2.0
HJHS ^1^ non-dominant knee	0.0	0.0–7.8
HJHS ^1^ total	29.0	16.5–40.8

^1^ HJHS: Hemophilia Joint Health Score.

**Table 2:** Leisure-time physical activity.

Frequency	N (%)
Never	0
<1 time/week	1 (7.14)
1 time/week	2 (14.29)
2–3 times/week	5 (35.71)
Almost daily	6 (42.86)
Intensity	
Take it easy	8 (57.14)
Push some	4 (28.57)
Near to exhaustion	2 (14.29)
Duration	
<15 min	0
16–30 min	1 (7.14)
30–60 min	7 (50.00)
>1 h	6 (42.86)
Resistance training	
Yes	10 (71.43)
No	4 (28.57)
Frequency	
1 time/week	3 (30.00)
2 times/week	4 (40.00)
3 times/week	2 (20.00)
4 times/week	1 (10.00)
Years of experience	
1 year	3 (33.33)
2 years	3 (33.33)
≥3 years	3 (33.33)
Intensity	
Moderate (60–70%)	9 (90.00)
Heavy (>80%)	1 (10.00)
